# Potential Therapeutic Action of Autophagy in Gastric Cancer Managements: Novel Treatment Strategies and Pharmacological Interventions

**DOI:** 10.3389/fphar.2021.813703

**Published:** 2022-01-28

**Authors:** Md. Ataur Rahman, Kazi Rejvee Ahmed, MD. Hasanur Rahman, Moon Nyeo Park, Bonglee Kim

**Affiliations:** ^1^ Department of Pathology, College of Korean Medicine, Kyung Hee University, Seoul, South Korea; ^2^ Korean Medicine-Based Drug Repositioning Cancer Research Center, College of Korean Medicine, Kyung Hee University, Seoul, South Korea; ^3^ Department of Biotechnology and Genetic Engineering, Global Biotechnology and Biomedical Research Network (GBBRN), Faculty of Biological Sciences, Islamic University, Kushtia, Bangladesh; ^4^ Department of Biotechnology and Genetic Engineering, Faculty of Biological Sciences, Islamic University, Kushtia, Bangladesh; ^5^ ABEx Bio-Research Center, East Azampur, Bangladesh

**Keywords:** gastric cancer, autophagy, phytochemical, tumorigenesis, autophagy-related genes, autophagy modulator

## Abstract

Gastric cancer (GC), second most leading cause of cancer-associated mortality globally, is the cancer of gastrointestinal tract in which malignant cells form in lining of the stomach, resulting in indigestion, pain, and stomach discomfort. Autophagy is an intracellular system in which misfolded, aggregated, and damaged proteins, as well as organelles, are degraded by the lysosomal pathway, and avoiding abnormal accumulation of huge quantities of harmful cellular constituents. However, the exact molecular mechanism of autophagy-mediated GC management has not been clearly elucidated. Here, we emphasized the role of autophagy in the modulation and development of GC transformation in addition to underlying the molecular mechanisms of autophagy-mediated regulation of GC. Accumulating evidences have revealed that targeting autophagy by small molecule activators or inhibitors has become one of the greatest auspicious approaches for GC managements. Particularly, it has been verified that phytochemicals play an important role in treatment as well as prevention of GC. However, use of combination therapies of autophagy modulators in order to overcome the drug resistance through GC treatment will provide novel opportunities to develop promising GC therapeutic approaches. In addition, investigations of the pathophysiological mechanism of GC with potential challenges are urgently needed, as well as limitations of the modulation of autophagy-mediated therapeutic strategies. Therefore, in this review, we would like to deliver an existing standard molecular treatment strategy focusing on the relationship between chemotherapeutic drugs and autophagy, which will help to improve the current treatments of GC patients.

## 1 Introduction

Gastric cancer (GC) is one of the most common frequently gastrointestinal and deadly cancers with more than one million new cases diagnosed yearly, which is the largest reason among the cancer fatalities worldwide (Hoang and Vu 2021). In the initial phages of GC, surgery is the most suitable option (Lin et al., 2021b). Because of its peculiar as well as cunning characteristics of initial diagnostic signs and symptoms, GC may result; even a minority proportion of cases are being accurately recognized, although more than 60% of patients had local or distant metastasis just at the moment of testing (Sun et al., 2021). Therefore, chemotherapy-mediated treatment seems to be preferred for the largest percentage of patients who belong to middle and late stage of GC (Xiao et al., 2021), although numerous patients are cured, but their rates of survival are very low. Principle of pathogenesis and progress of GC cancer is also mostly unclear (Tang et al., 2021). Because of drug resistance, patients with GC often have no sensitivity to chemotherapy, which is the primary reason of chemotherapeutic failure and poor success probability (Riquelme et al., 2016).

Autophagy, engulfing dysregulated organelles and cellular macromolecules, is an evolutionarily preserved catabolic active process concerning the formation of autophagosomes, leading to breakdown of cellular constituents after fusion with lysosomes (Rahman and Rhim 2017). In addition, cellular degradation procedures mostly fall into two classes, macroautophagy, which is commonly known as autophagy, and ubiquitin–proteasome system (Rahman et al., 2020c; Uddin et al., 2020). Furthermore, autophagy regulates in the activation of several cancer-related genes, which inhibits tumor promotion and suppression (Botti et al., 2006; Maiuri et al., 2009). Recent studies have widely explained the involvement of autophagy in GC growth, metastasis, and forecasting (Xu et al., 2020a; Spirina et al., 2020; Xiu et al., 2021). Moreover, microRNAs (miRNAs), short (∼22 nucleotides in length) noncoding RNA molecules, can control gene expression at a posttranscriptional level, which has an important association in autophagy-mediated GC regulation. There are abundant convincing studies that showed inseparable association between miRNAs and GC (Shao et al., 2020). miRNAs affected GC, which includes oncogenesis, diagnosis, development, treatment, and prognosis, although many miRNAs have been linked to GC, and few could be useful to clinical practice (Ouyang et al., 2021), even though numerous miRNAs have been related to GC and few can be applied to clinical practice as well. Furthermore, diet-related natural ingredients may control autophagy in the GC cell that promote cancer cell chemosensitivity (Xu et al., 2020a). In the present study, we would like to represent an overview of the literature in association with autophagy modulation in GC treatment as well as the role of autophagy boosting and suppressing, which control GC growth and invasion as a potential treatment strategy and managements.

## 2 Molecular Mechanism of Autophagy Regulation and Cancer Progression

**FIGURE 1:**
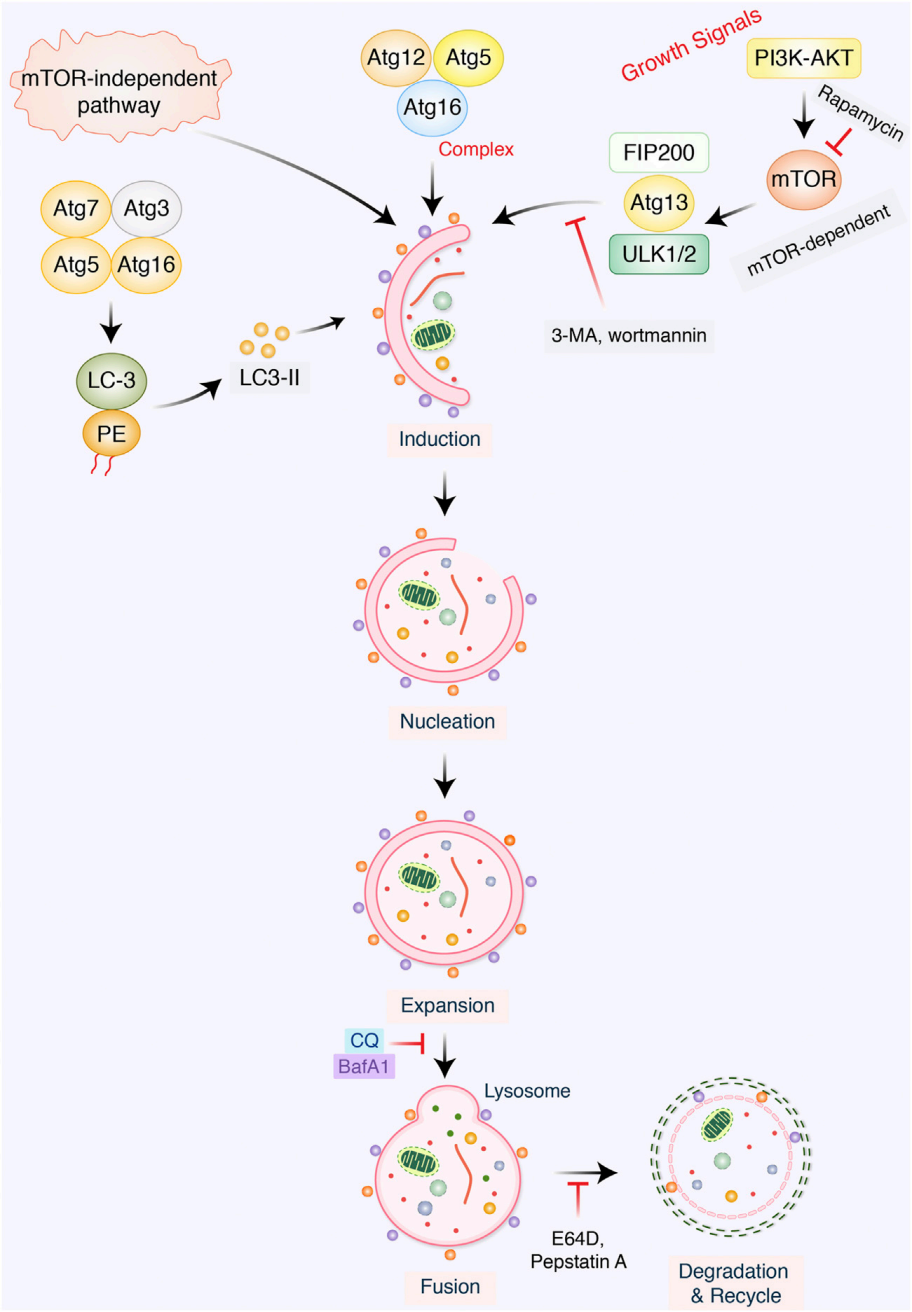
Mechanism of the autophagic pathway. Autophagy initiates *via* the formation of a macromolecular assembly structure. PI3K-AKT and mTOR contribute to the formation of the phagophore assembly site (PAS). ULK1/2, ATG-13, Vps34, and beclin-1 complex activate phagophore formation, which creates nucleation elongation as a result of autophagosome formation. Mature autophagosome and lysosome bind to form autolysosome formation. Eventually, autolysosomes are degraded *via* acid hydrolases, thereby releasing nutrients as well as recycling metabolites.

Autophagy is a self-digestion process that assists in maintaining cellular homeostasis through recycling unwanted or damaged toxic cellular organelles into the cells (Rahman and Rhim 2017; Rahman et al., 2021b). Autophagy modulation has been implicated to regulate several cancers and neurodegeneration (Rahman et al., 2020b; Rahman et al., 2020d). Generally, autophagy process might be introduced *via* the accumulation of preautophagosome structures formation, which is known as phagophore assembly sites (PASs) (Hurley and Young 2017; Rahman and Rhim 2017). Phosphatidylinositol 3-phosphate PI3K associated with endoplasmic reticulum (ER) has an essential role to imitate PAS formation (Kotani et al., 2018). AMPK, AMP-activated protein kinase, mTOR, mammalian target of rapamycin, and ULK1, unc-51 like autophagy activating kinase-1, have been facilitated to initiate phagophore formation in the process of autophagy induction (Alers et al., 2012). However, beclin-1, Vps34, and Vps15/p150 help to recruit formation of phagophore (Velazquez and Jackson 2018). After that, phagophore nucleation has been followed to elongate membrane to form autophagosome formation (Rubinsztein et al., 2012). Mature autophagosome binds to lysosome, which results to autolysosome formation (Kardideh et al., 2019). Finally, autolysosomes that contain inner cargos have been degraded by acid hydrolases, as well as produce nutrients and other recycling metabolites, resulting to maintenance of intracellular homeostasis inside the cells (Figure 1).

A direct connection has been found between autophagy and cancer (Liang et al., 1999). Recently, a huge number of researches have indicated that ATGs as well as associated pathways may crosstalk between oncogenes and tumor suppressors (Ariosa et al., 2021; Khaleel 2021). Certainly, collected data have supported that the role of autophagy in cancer is complicated, which may have opposite values in a context- as well as cell type–dependent manner (Li et al., 2021b). Autophagy determines whether the cancer is inhibited or activated under certain conditions. It has been mentioned that mTOR plays a central role in activating or protecting oncogenic cells *via* induction of autophagy (Xu et al., 2020b). In addition, inhibition of autophagy pathway may regulate cancer progression, as well as the influence of autophagy develops into either a death function or cellular survival function (Jung et al., 2020). The metabolism of cancer cells is strongly changed to retain their survival and proliferation under adverse microenvironmental situations. It was found that autophagy acts as an essential function in maintaining metabolic variations in cancer cells (Goldsmith et al., 2014), although autophagy is familiar to sustain neoplastic cell metabolism during stress, and the mutual relation between cancer cell metabolism and autophagy remains unknown. AMPK and mTOR have been recognized as the enteral signaling mechanisms which regulate autophagy through the regulation of amino acid as well as glucose levels (Alers et al., 2012). However, the specific metabolites, oxygen concentration, growth factors, ROS, ATP-to-ADP ratio, specific amino acid levels, palmitate, and oncogenes regulate autophagy initiation in addition to autophagosome formation. In addition, they regulate the balance *via* assimilating the autophagy-related signals in cancer (Singh and Cuervo 2011; Panda et al., 2015). Conspicuously, autophagy has been commonly recognized to play a “double role” as it can either delay or activate cancer initiation as well as progression (Rahman et al., 2020b; Patra et al., 2020).

**FIGURE 2:**
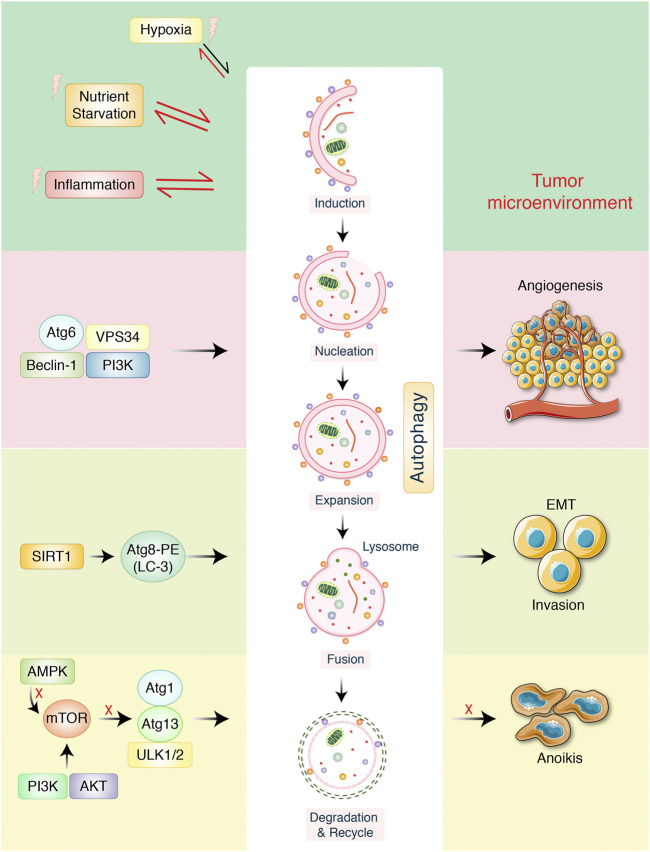
Autophagy-mediated metastasis formation in gastric cancer. The autophagy-related proteins are involved in regulation in cancer. Starvation, hypoxia, and inflammation might be stimulated during autophagic process which create tumor microenvironment. VPS34, ATG6, beclin-1, and PI3K increase tumor angiogenesis. Transcription factor SIRT1 activates autophagy improvement *via* inducing ATG8-LC-3-PE conjugation, which later encourages epithelial-to-mesenchymal transition (EMT), as well as tumor invasion. mTOR negatively regulates autophagy *via* inhibiting ATG13-ATG1-ULK1/2 protein complex. AMPK/PI3K regulates autophagy, which detached tumor cells to overcome anoikis.

The dual role of autophagy in cancer has been emphasized in autophagy regulation as well as cancer cell activation, which metabolism controls tumor growth and progression. Basically, the actual role of autophagy in GC prognosis, metastasis, and progression has not been widely deliberated yet. However, tumor metastasis sign has predicted advanced progression as well as poor prognosis of GC (Jin et al., 2014: ; Cai et al., 2020). Tumor metastasis process is complex, involving a series of pathological actions, for example, breakdown of epithelial-to-mesenchymal transition (EMT), extracellular matrix (ECM), tumor microenvironment formation, and tumor angiogenesis (Winkler et al., 2020). In addition, in tumor metastasis, the role of autophagy is supposed to be both prometastatic and antimetastatic effects (Poole and Macleod, 2021). Concerning GC, while autophagic cell death may prevent metastasis, most of the current results support the notion that autophagy facilitates tumor metastasis *via* affecting several aspects (Figure 2). In the present study, we would like to focus on the relationship with chemotherapeutic treatment of GC through modulation of autophagy pathway.

## 3 Role and Regulation of Autophagy in GC Management

During gastric carcinogenesis, the complicated autophagy-mediated regulated pathways become more complicated, and it is necessary to properly investigate. Primarily, AMPK regulatory system interacts with PI3K-AKT signaling, although a multitude of transcription components regulate cell biological activities such as multiplication, development, apoptosis, and autophagy in anticancer activities. Clinical and pathological studies have shown that tumor tissues have lower levels of AMPK expression than normal tissues, and this is thought to be a contributing factor to emergence and progression of melanomas (Faubert et al., 2015; Scazzone et al., 2021). Perillaldehyde activates AMPK by phosphorylating LKB1 at the S307 and S428 regions, and AMPK induces GC cell autophagy by phosphorylating and activating ULK1, which restricts GC cell proliferation (Zhang et al., 2019). MTDH also performs a vital role in drug resistance, specifically in the resistance to 5-fluorouracil (5-FU), doxorubicin, CDDP, and etoposide, but also paclitaxel. MTDH governs ATG-5 expression through triggering AMPK phosphorylation, recommending that MTDH may invoke autophagy *via* the AMPK/ATG-5 signal transduction pathway and encourage drug tolerance in GC cells (Pei et al., 2018). Cancer cells with an abnormally active PI3K/AKT/mTOR pathway have a higher propensity to proliferate aggressively, extending their survival period and becoming resistant to chemotherapy (Zheng et al., 2021). A study has demonstrated that activating AKT makes GC cells more resistant to chemotherapy treatments such 5-FU, doxorubicin, mitomycin C, and cisplatin (5-FU) (Munshi et al., 2001). Autophagy is inhibited by the mTOR route, which negatively regulates; therefore, blocking the mTOR system is essential to triggering autophagy (Yap et al., 2008). Flavonoids can act as an anticancer element by inhibiting the PI3K/AKT/mTOR system, resulting in arresting G2/M cell cycle and autophagy, which induce death of GC cells (Raha et al., 2015). Furthermore, it has been shown that suppressing YWHAZ in BGC-823 cells inhibits the PI3K/AKT/mTOR regulatory mechanism, resulting in cell death and autophagy (Guo et al., 2018a). lncRNAs-HAGLROS associates mTORC1 that stimulate the mTORC1 regulatory system, boosting GC cell proliferation and sustaining its malignant state (Chen et al., 2018a). Furthermore, elevated production of HAGLROS correlates with the formation and lousy prediction of GC, according to new research findings (Chen et al., 2018a). A number of investigations suggested that long noncoding RNAs (lncRNAs) influence chemoresistance in a variety of cancers (Fan et al., 2014; Qu et al., 2016; Özeş et al., 2016). Moreover, ARHGAP5-AS1 is a novel drug-resistant lncRNA that increases in GC-resistant cells and can revert chemoresistance and afterward turned down (Zhu et al., 2019).

Autophagy is thought to be a beneficial process for inhibiting tumor development at numerous phases, conserving genetic consistency, removing intracellular supplies of reactive oxygen species (ROS), as well as sustaining bioenergetic activities (Wang et al., 2018; Weng et al., 2018). Autophagy, as both a cell’s stress feedback system to inside and outside stimuli as well as promote cellular damage also the life expectancy of cancer cells subjected to chemotherapeutics. (Scherz-Shouval et al., 2010). Autophagy separates cellular components for example mitochondria inside of cells, that also helps stop the propagation of pro-apoptotic elements inside of cells and thus aids the tumor type of cells avoiding apoptosis. Autophagy suppression in cancerous cells could also increase the toxic effects of anti-tumor prescription medications and backward drug resistance (Kumar et al., 2015; Belounis et al., 2016). Autophagy and apoptosis can be induced by apatinib and astragalus polysaccharides, however this polysaccharide inhibit metastasis of GC and invasion of GC. Whilst autophagy blockers could even significantly boost AGS apoptotic cell death, it appears that such increased autophagy caused by apatinib safe guards the cells from apoptosis. Because increased autophagy could have negative impacts on chemo, inhibiting autophagy can stimulate protective GC cells and improve the anti-tumor influence of chemotherapeutics (Wu et al., 2018b). Autophagy suppression appears to have a pro-apoptotic impact on peoples GC cells. Cinobufagin can cause the generation of ROS which causes apoptotic cell death as well as autophagic cell death through stimulating the ROS/JNK/p38 alignment. Steadily increasing proapoptotic protein expression, abnormal mitochondrial membrane potential, and elevated ROS manufacturing are observed while autophagy is interrupted, implying that autophagy suppression improves cinobufagin-induced cell death, which might take place in part *via* the mitochondrial-coded cell death passageway (Xiong et al., 2019).

Autophagy could indeed preserve cell equilibrium in the preliminary phases of tumorigenesis, inhibiting the incidence as well as advancement of GC. Autophagic cell death is distinct from apoptotic cell death. Autophagy and apoptosis coexist in GC cells, and their interplay governs cell death autonomously. Apoptosis occurs downstream of autophagy, as well as apoptotic cell death that occurs by autophagy (Liu et al., 2021a). For instance, the PI3K/AKT/mTOR sensing process, which can synchronously govern the destiny of GC cells, can control autophagy and apoptosis, respectively (Hu et al., 2021). Caffeine and theophylline, which are derivatives from methylxanthine, have been shown to suppress the PI3K/AKT/mTOR paths through activating PTEN. As a result, GC cells undergo apoptosis and autophagy, and their expansion is inhibited (Liu et al., 2019). ER stress and its unfolded protein response can also be linked to GC cell ability to survive, advancement, and medication resistance *via* biological processes such as autophagy. Melatonin induces cell autophagy *via* ER stress, helping to promote GC apoptotic cell death and preventing their growth, expansion, and invasion (Liu et al., 2019; Peng et al., 2019). Mitochondrial apoptosis can be induced by tetrandrine and also inhibit the AKT/mTOR pathway in HGC-27 cells causing autophagy and apoptosis, resulting in antitumor action and death of cells in gastric tumors. During the tetrandrine-induced antitumor procedure, autophagy and apoptosis work together to improve tumor cell death (Bai et al., 2018). Beclin-1 also serves as an important role enhancing autophagy-induced apoptosis resistance, and the expressions of beclin-1, Bcl-xl, and Bcl-2 are positively correlated with autophagy (Menon and Dhamija 2018). Beclin-1 can also stimulate the expression of Bcl-2 and Bcl-xl; an alternative is to suppress Bak and Bax protein levels while increasing levels of cleaved caspase in GC cells (Kang et al., 2011). This will prevent GC cells from undergoing apoptosis while boosting autophagy in GC cells (Maiuri et al., 2007; Du and Ji 2014).

## 4 Autophagy Markers Expressed in GC Progression

Because of the differences in biological and clinical characteristics, carcinoembryonic antigen and carbohydrate antigen 19-9 have been found to be the most common GC markers measured before and after surgery (Lin et al., 2020), although which preoperative or postoperative combined tumor markers have a more prognostic value has not been clear yet, as well as whether change of the preoperative and postoperative systemic inflammatory response (SIR) levels affects the prognosis of GC. Perioperative SIR variations were described as changes in the neutrophil–lymphocyte ratio, lymphocyte–monocyte ratio, systemic immune–inflammation index, and platelet–lymphocyte ratio (Lin et al., 2021c). Autophagy-mediated tumor metastasis in GC is described in Figure 2, which is modified from Qian and Yang in 2016 (Qian and Yang 2016). Inhibition of mTOR by cellular energy sensor AMPK activates autophagy, which acts a prosurvival role in cancer cells during ECM detachment (Mukhopadhyay et al., 2021). Improved autophagic process might prevent ECM detached cells from anoikis, in addition to contributing to luminal filling probably through providing sustained ATP sources (Santagostino et al., 2021). Therefore, autophagy has been considered as an adaptive strategy for separate cancer cells to overcome anoikis in the early stage of cancer progression (Rahman et al., 2020d; Ariosa et al., 2021). Besides, a class III histone deacetylase, silent mating type information regulation 1 (SIRT1), is augmented in tumor tissues, in addition to correlating with metastasis of GC in advanced lymph node (Mody et al., 2021). However, the regulatory effects of SIRT1 in EMT, as well as invasion ability of GC, were further confirmed by an *in vitro* study (Xu et al., 2021a). Significantly, it has been found that SIRT1 is a well-categorized autophagy mediator that is derived to initiate autophagy through deacetylation of LC-3 ATGs (Chen et al., 2021a). Collecting this observation, autophagic process is modulated *via* SIRT1 or other mediators that might play an essential role in tumor progression *via* modulating EMT as well as tumor cell invasion (Zia et al., 2021). Recently, it has been reported that autophagy encouraged the survival as well as invasive capability of SGC-7901 cells *via* facilitating the development of vasculogenic mimicry (Ahmadpour et al., 2021). Particularly, autophagy inhibition by beclin-1 silencing expression decreased cancer cell invasion and survival (Ding et al., 2014). Therefore, beclin-1–mediated autophagy might be considered as a tumor angiogenesis factor for GC. Pharmacologically, autophagy inhibition along with antiangiogenic therapy may combine as a promising way to overcome tumor angiogenesis. Moreover, concerning the tumor microenvironment, autophagy might be strongly encouraged *via* nutrient depletion, hypoxia, and inflammation (Poillet-Perez et al., 2021). However, it is also mentioned that activated autophagy shapes the tumor microenvironment through activating tumor angiogenesis, regulating inflammatory responses, and providing nutrient supply (Mukhopadhyay et al., 2021).

Based on The Cancer Genome Atlas database, gene expression data for GC patients undergoing numerous other molecular markers have been recognized to possess prognostic value and their expression patterns of autophagy-related genes (ATGs) in GC cells, which served as a hallmark of autophagy regulation (Liu et al., 2021b). Autophagy and GC are controlled by crucial kinases such as mTOR, PI3K/AKT, AMPK, and MAPK, as well as epidermal growth factor receptor, cell cycle mediators, vascular endothelial growth factor, cytokines, apoptosis-associated regulators, and miRNAs (Xiu et al., 2021). ATG5 has been found to participate in autophagosome elongation as a vital regulator and is crucial for autophagy, which is associated with chemotherapy resistance of maximum cancer cells (Wang et al., 2019). Moreover, single-strand conformation polymorphism analysis has been revealed that the frameshift mutations in ATG genes with mononucleotide repeats, including ATG-2B (mammalian ATG-2 homolog), ATG-9B (mammalian ATG-9 homolog), and ATG-12 have in common GC with high microsatellite instability subtypes (Kang et al., 2009). ABCC1 encodes the multidrug-resistant protein 1 (MRP1), which promotes the MDR phenotype in GC. However, MRP1 and ATG5 expression were found to be positively associated and ATG5 expression can sometimes lead to more forceful and malignant trait of GC, which could provide important data for effectively evaluating chemotherapy impact in GC patients (Xu et al., 2020a). ATG5 and MRP1 expressions are being worked as self-governing prognostic markers for predicting overall survival and disease-free survival in GC patients (Ge et al., 2014). A rate-limiting enzyme arginine synthesis pathway, arginine succinate synthase 1 (ASS1), is highly expressed in GC tissues (Silberman et al., 2019). Studies have found that ASS1 may be a useful prognostic marker for predicting survival and metastasis in patients with GC (Tsai et al., 2018; Jangra et al., 2020). The gene SP1, part of the SP1 multigene family, which is critical in the emergence and progression of malignancies, was found to be highly overexpressed in GC tissues, and this overexpression was closely associated with patient survival (Xu et al., 2018). SP1 has an adverse effect on autophagy regulation because it binds directly to the p62 promoter and raises p62’s expression level. As the SP1-p62 axis may contribute to the development of GC, it could serve as a prognostic indicator for the detection of GC (Xu et al., 2018). Moreover, newly synthesized LC-3’s C-terminus has been hydrolyzed *via* a cysteine protease known as ATG-4B exposing Gly-120 called LC-3-I (Agrotis and Ketteler 2020). LC-3-I has been processed *via* a series of ubiquitin-like reactions by the help of enzymes ATG3, ATG7, and ATG12–ATG5–ATG16, which become adjacent to the head group of the lipid phosphatidylethanolamine (PE), a class of phospholipids found in biological membranes (Rahman et al., 2021a). However, the lipid modified form of LC-3, which is known as LC-3-II, has been believed to be intricated in autophagosome membrane elongation as well as fusion events during autophagy process (Schaefer and Dikic 2021). However, the exact function and role of LC-3 in autophagy pathway are still investigated. In addition to the PB1 domain and the TB domain, p62, also known as sequestosome 1 (SQSTM1) autophagosome cargo protein, which targets other proteins that bind to it for selective autophagy, has several other domains such as the KIR, a ubiquitin-related area, and so on. A number of investigations have revealed that p62 levels are inversely associated with levels of GC autophagy (Weng et al., 2018). When autophagy occurs, the expression of beclin-1 increases because it is a yeast ATG6 homolog and is an essential activator of autophagy (Hu et al., 2015), and a reduction in beclin-1 expression in GC indicates a drop in autophagy (Zheng et al., 2019).

## 5 Role of miRNAs Through Regulation of Autophagy in GC

**FIGURE 3:**
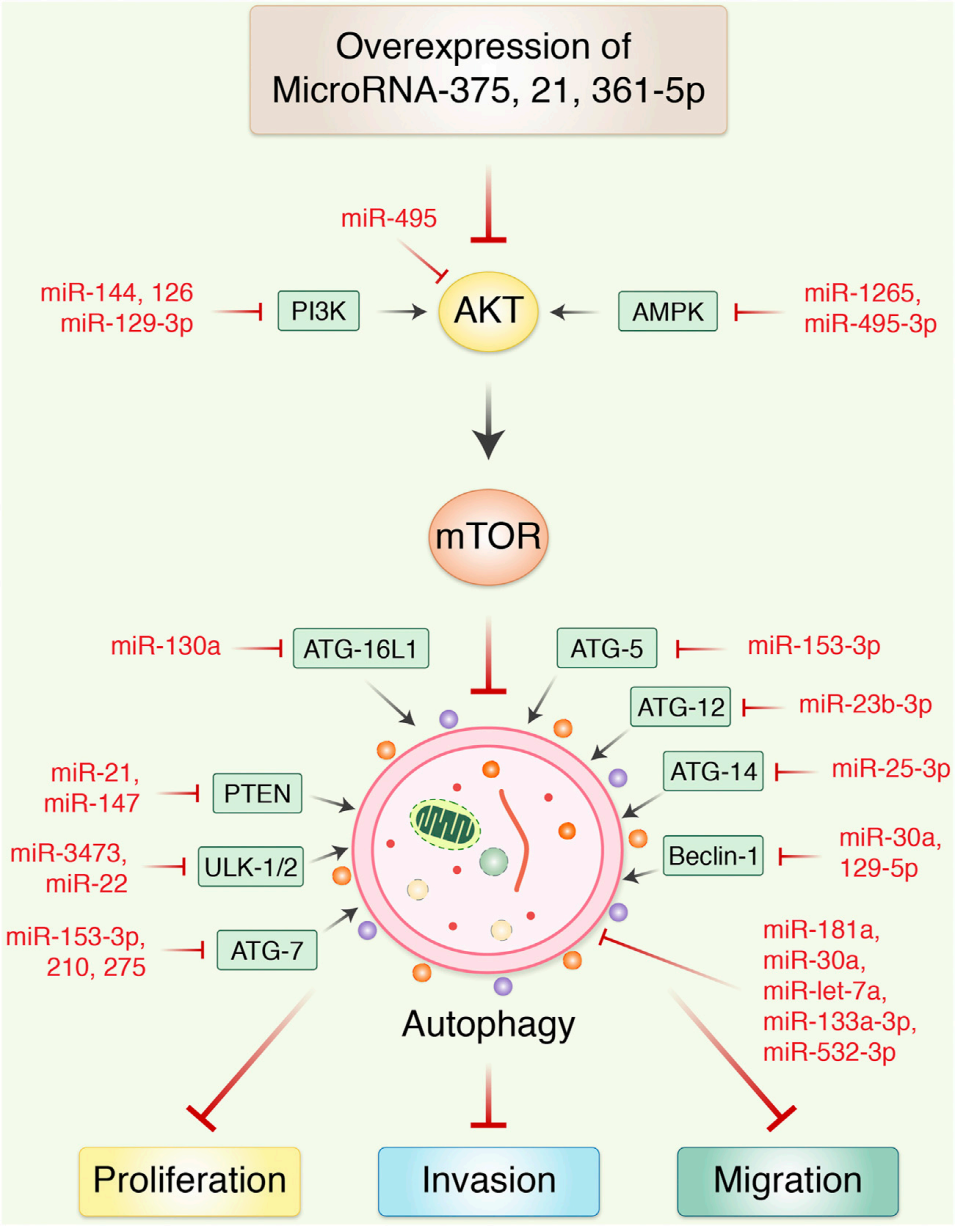
MicroRNAs regulates autophagy-mediated cell proliferation and migration in gastric cancer. Overexpression of miR-375 inhibited the proliferation and migration of gastric cancer *in vitro* and xenograft nude mouse model. miRNA blocks autophagy *via* AKT/mTOR signaling pathway and regulating invasion as well as migration in epithelial-to-mesenchymal transition. In addition, most usually effective miRNAs control the transcriptional expression of upstream activators and inhibitors of autophagy in gastric cancer.

**FIGURE 4:**
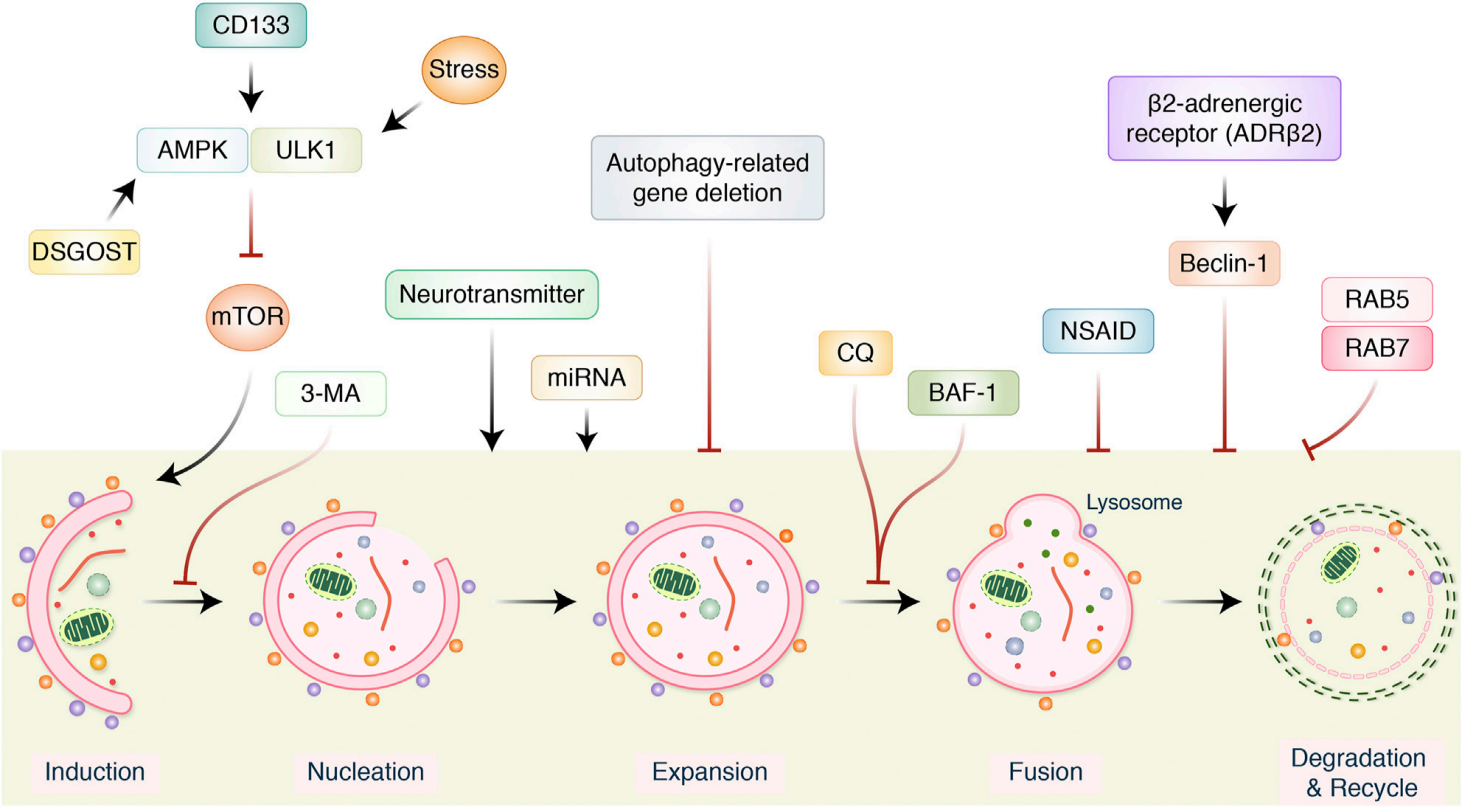
Several signaling pathways modulating autophagy in gastric cancer treatment. AMPK/ULK1 inhibits mTOR pathway, which positively activates autophagy induction. Neurotransmitter and miRNA regulate autophagy induction. However, autophagy-related gene (ATG), 3-metheylalanine (3-MA), chloroquine (CQ), bafilomycin A1 (BAF-1), and nonsteroidal anti-inflammatory drugs (NSAIDs) inhibited and modulated entire autophagy process. β_2_-Adrenergic receptor activates beclin-1 and inhibits autophagy.

Alongside miRNAs, lncRNAs are essential to regulate autophagy, which can aid in the development of more effective treatment strategies and the identification of novel medicinal aims for the research of processes of resistance in GC (Roy 2021). In GC tissues, lncRNA MALAT1 activates autophagy *via* downregulating the tumor suppressor miR-204. miR-204 overexpression in GC cell lines CTC105 and CTC141 reduces transient receptor potential melastatin 3 (TRPM3), an activator of oncogenic autophagy-miR-204, which can be used as a target in GC therapy. Antiautophagy tumor suppressor, miR-30a, upsurges sensitivity to imatinib (IM) in gastrointestinal stromal tumor (GIST) cells alone with mouse xenograft models with specific target of miR-30a in association with beclin-1–driven autophagy in IM-resistant cells such as GIST-882 than the sensitive GIST-1 cell line (Shao et al., 2020), but counterintuitive that miR-183 could contribute to GC suppression (Li et al., 2019). Furthermore, miR-20a, an oncogene, is significantly expressed in GC, and it is anticipated that it will serve as an indicator for clinical identification (Xin et al., 2019). It has been found that miR-21 is overexpressed, and its anomalous expression has an important role in GC growth *via* modulating tumor suppressors PTEN and PDCD4 expression, which regulates migration, cell growth, invasion, and apoptosis (Li et al., 2014). According to the findings, the level of miR-1265 in GC samples was shown to be lower than in samples from nearby healthy cells. The calcium-binding protein (CAB39) gene is miR-1265’s target. CAB39 is an important element of the LKB1-STRAD-CAB39 combination (Brajenovic et al., 2004; Treiber et al., 2019), autophagy produced by the CAB39-LKB1-AMPK pathway is cancerous in GC cells and enhanced the phosphorylated of AMPK more than 100-fold (Hawley et al., 2003) at the Thr172 junction of LKB1 with STRAD and CAB39. As a result, miR-1265 slows GC development and autophagy by reducing the expression of CAB39 and controlling the AMPK-mTOR regulatory pathway (Xu et al., 2019). On the other hand, targeting miR-25-3p activated growth inhibition, invasion, and migration of GC cells *in vitro*, and *in vivo* delivery of miR-25-3p inhibitors significantly encouraged SCID mice–bearing human GC xenografts antitumor activity (Ning et al., 2020). An miRNA, miR-495-3p, has been linked to the development of malignant phenotypes in patients with GC. It is thought to be responsible for reversing MDR by inhibiting autophagy through modulation of the mTOR signaling pathway. MiRNAs-181a endorses EMT in esophageal squamous cell carcinoma through the transforming growth factor-β/Smad pathway (Xu et al., 2021b). It is also believed that miR-495-3p is responsible for reversing MDR by limiting autophagy and also that miR-495-3p is related to the malignant phenotype of GC patients (Chen et al., 2018b). In addition, certain miRNAs, such as miR-375 (Yuan et al., 2018), miR-21 (Gu et al., 2020), and miR-361-5p (Tian et al., 2018), which operate as autophagy blockers, control autophagy and limit GC activity *via* modulating the mTOR pathway (Figure 3 and Figure 4). Furthermore, the expression of miRNAs, such as miR-181a (Zhao et al., 2016), miR-30a (Du et al., 2018), miR-let-7a (Fan et al., 2018), miR-133a-3p (Zhang et al., 2018a), miR-532-3p (Guo et al., 2018b), and others, is connected with such a reduction in the capacity of GC cells to proliferate. It has been found that SLC7A11 is identified as a target of miR-375, which diminished the stemness of GC cells *via* activating SLC7A11-dependent ferroptosis (Ni et al., 2021). MALAT1, a competing endogenous RNA of miR-23b-3p, was shown to reduce the suppressive activities of miR-23b-3p on ATG-12, while simultaneously increasing the development of ATG-12, resulting in chemoinduced GC cell autophagy and drug resistance (YiRen et al., 2017). In addition, AKT and mTOR have been reported to be targeted *via* miR-495 overexpression of miR-495, which could prevent the growth in addition to induce the apoptosis of GC cells through blocking of PI3K/AKT/mTOR, which altered Bax, caspase-3/-9, and cyclin D1 expression (Ouyang et al., 2021). miR-153-3p facilitates ATG-7–mediated autophagy induction in fluorouracil resistance *via* the adenosine monophosphate (AMP)-activated protein kinase (AMPK)/ATG3 pathway in GC (Hou et al., 2020). Gastric carcinogenesis is complicated by the participation of miRNAs (Song and Meltzer 2012), and these autophagy-mediated processes must be further investigated.

## 6 Therapeutic Target and Treatment Strategy of Autophagy Modulation in GC

Therapeutic targeting of autophagy in GCs might be proposed to be an auspicious novel therapeutic approach. Meanwhile, both autophagy inhibitors and autophagy inducers may lead to inhibit cancer cell death, but at present, they are only in the clinical development stage for the treatment of GCs. Despite the fact that the association between autophagy and cancer still is debated, the participation of shared regulatory mechanisms (Table 1) makes autophagy an attractive therapeutic focus for the treatment of cancer (Khaleel 2021). Autophagy can be used to sensitize tumor cells to chemotherapy or radiation, therapy blocking its cytoprotective action, which leads to the destruction of antiapoptotic cells *via* autophagy (Gupta et al., 2021). Through the use of their mechanisms, autophagy demonstrated resistance to chemotherapy and radiotherapy, and it also hinders the effectiveness of anticancer medicine (Zhang et al., 2021). Inhibiting autophagy in cancerous cells can have adverse repercussions on the body, including such mitochondrial dysfunction, redox imbalance, nucleotide consumption, and reduction of energy supply (Poillet-Perez et al., 2021). As a result, inhibiting autophagy could be recruited to increase chemosensitivity as a therapeutic strategy. AMP-activated protein kinase *α* (AMPK*α*) has been shown to promote autophagy by triggering ULK1 and blocking mTOR/p70S6K process (Xu et al., 2020b). Stress-inducing neurotransmitter norepinephrine increases autophagic flux by AMPK-ULK1 pathway, as a result activating the tumor-promoting autophagy pathways and speeding up GC development (Zhi et al., 2019). Anticancer therapy efficacy may be improved using AMPK inhibitors; 3-methyladenine (3-MA), a class III PI3K inhibitor, suppresses autophagy by inhibiting autophagy from initial stage and preventing autophagosomes formation. Antimalarial medication chloroquine (CQ) is used to treat malaria, which also impedes autophagy (Lin et al., 2021a). There is no direct impact on organelle acidity (Park et al., 2021), but it suppresses autophagy by preventing the autophagosome and lysosome from joining (Honma et al., 2021). The effectiveness of antitumor therapy could be improved by using this supplement; CQ and 5-FU together have the potential to further reduce the number of GC stem cells (Xiao et al., 2017). As a result of blocking lysosome–autophagy fusion, BAF-1 shows lower LC-3-II levels while simultaneously elevating p62, which is one way that BAF-1 inhibits autophagy (Bai et al., 2018). However, there is no information on the connection between BAF-1 and the therapy of GC. In addition, chemokine CXCL12 encouraged mTOR activation and played an important role in GC cell peritoneal metastasis control (Mele et al., 2020).

**TABLE 1:** Several molecular target and therapeutic role of different drugs in autophagy modulation in gastric cancer cells.

Compounds	Experimental model	Autophagy mechanism	References
Chloroquine	Bone marrow stromal cells	Autophagy induction	Lin et al. (2021a)
Nonsteroidal anti-inflammatory drugs	SGC-7901 cells	Autophagy induction	Fletcher et al. (2021)
3-Methylalanine	SGC-7901 cells	Class III PI3K inhibitor and suppresses autophagy	Li et al. (2013)
Bafilomycin A1 (BAF-1)	MGC-803 cells	Autophagy induction	Bai et al. (2018)
β_2_-Adrenergic receptor	Human SGC-7901 and BGC-823 cells	AMPK-ULK1 mediated autophagy induction	Zhi et al. (2019)
Indomethacin	AGS cells	Lysosomal-mediated autophagy induction	Ahmed et al. (2021)
CXCL12	NUGC4 cell	mTOR-mediated autophagy activation	Mele et al. (2020)
Compound C	AGS cells	Inhibitory role of autophagy	Li et al. (2013)
Rapamycin	Human SGC-7901 and BGC-823 cells	Induction of autophagy	Chen et al. (2021b)

Recently, it has been found that inhibition of autophagy *via* silencing of beclin-1 protein expression decreased GC cell survival as well as invasion (Shafabakhsh et al., 2021). The deletion of ATGs then suppresses autophagic activity *via* siRNA- or miRNA-mediated silencing methods, which have been gaining attention (Schaefer and Dikic 2021). The use of sequence-specific DNA or RNA analogs can be used to build customized compounds with anticancer properties at a low cost that prevent the production of specific gene sequences with high specificity. In addition, several essential controllers of autophagy pathways, such as ATG-3, ATG-4B, ATG-4C, ATG-5, ATG-6, beclin-1, ATG-10, and ATG-12, can be targeted in this way in order to combat GC (Mandhair et al., 2021). It has already been discovered that enhanced ATG-5 activity was observed to be associated with a better patient survival and disorder survival in GC patients and that ATG-5 was abundantly expressed in drug-resistant GC cell lines. It has also been discovered that suppressing ATG-5 (siRNA-ATG-5-695) can restore sensitivity to chemotherapeutics in resistant cells (Xu et al., 2020a). GC cells treated with cinobufagin undergo apoptosis because of the inhibition of ATG-5 synthesis caused by siRNA, which can also increase ROS formation and activate a cell death pathway in mitochondria. The stimulation of autophagy in GC cells by norepinephrine is a critical component for the growth of GC (Krizanova et al., 2016; Le et al., 2016; Zhi et al., 2019). The β_2_-adrenergic receptor (ADRB2) is a variant of the adrenergic receptor that is responsible for catecholamine production in the body. Beclin-1 production can also reduce by deletion of the ADRB2 gene that may also inactivate the AMPK-ULK1 system; as a result, autophagy is reduced (Xiu et al., 2021). When it comes to the autophagy function, Rab5a, a part of the Rab group, is also engaged in intracellular material transport and protein classification, but it also plays a role in the process (He et al., 2021). In GC cells, activation of mTOR by Rab5a, an upstream regulator of mTOR, can suppress autophagy and increase pharmacological resistant through activation of mTOR (Li et al., 2021b). Because of this, a Rab5a mutation can reduce mTOR activity in SGC7901 cells while simultaneously increasing autophagy and reversing pharmaceutical tolerance (Li et al., 2021a). Nonsteroidal anti-inflammatory drugs (NSAIDs) target the epithelium of the gastrointestinal system (Fletcher et al., 2021). If these medications are used to treat inflammation and pain, they have a detrimental effect on the digestive epithelia, which is their primary negative impact (Fratter et al., 2021). However, because NSAIDs decrease carcinogenesis in gastrointestinal tissues, they are considered an adjuvant to chemotherapy (Wang et al., 2021). It has been demonstrated that indomethacin-treated AGS cells exhibit decreased lysosomal acid content and increased membrane permeability, impairing lysosomal role and cathepsin action; thus, inadequate deterioration of autophagic materials impairs autophagic flux, raising the susceptibility of GC cells to cytotoxic agents (Ahmed et al., 2021). Rapamycin increases intracellular ROS generation, as well as displays selective synergistic antitumor activity with EF24 in human GC cell lines SGC-7901 and BGC-823 (Chen et al., 2021b). Therefore, perturbation of autophagic modulation may be a possible approach for controlling and treating GC.

## 7 Phytochemicals for the Prevention and Treatment of GC *via* Autophagy

**FIGURE 5:**
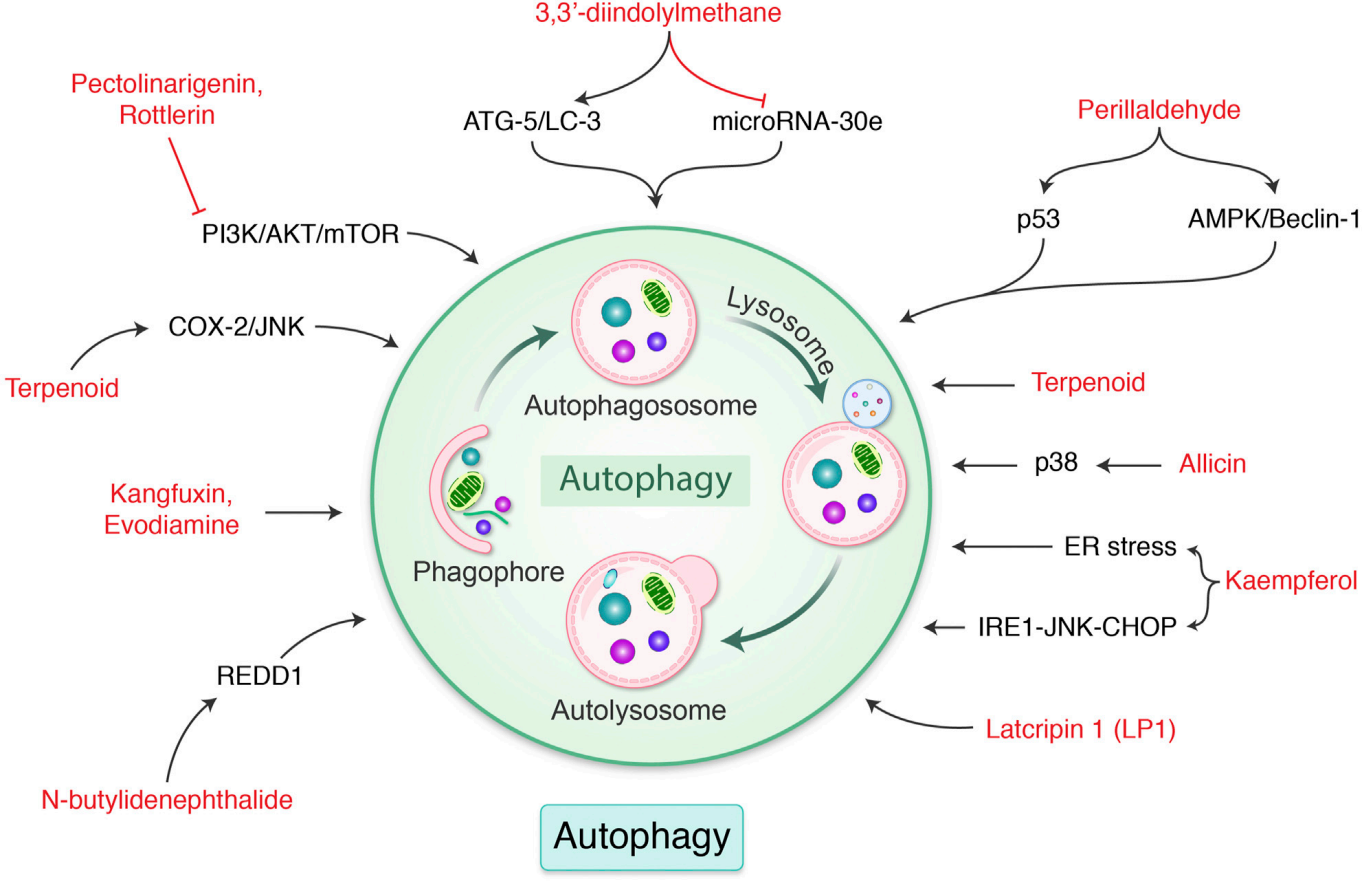
Phytochemicals modulate autophagy-mediated cell death in gastric cancer. Different naturally occurring molecules are regulated ER stress, p38, p53, ATG gene, COX-2, and mTOR pathway, which modulate autophagy in gastric cancer. Each compound is well-described in the text.

Phytochemicals have been demonstrated to be promising for regulating and controlling GC (Mitra and Dash 2018), which makes it possible for cellular components to degrade and be recycled in a controlled manner (Yao et al., 2021). Numerous phytochemicals and their autophagic activities are summarized in Table 2. Rottlerin, extracted from *Mallotus philippensis* Muell (Euphorbiaceae), induced autophagy and caspase-independent apoptosis against SGC-7901 and MGC-803 cells (Song et al., 2018). Evodiamine activates autophagy through beclin-2 expression in SGC-7901 GC cells (Rasul et al., 2012). *Morus alba* root extract, containing oxyresveratrol, has been found to accumulate ROS production and initiate autophagic and apoptotic cell death *via* FOXO-caspase-3 pathway (Kwon et al., 2015; Rahman et al., 2017). Cytotoxic activity on AGS, MKN-45, and KATO-III human GC cells *via* induction of caspase activation and autophagy *via* AKT/NF-κB pathway in AGS cells (Kang et al., 2021) have been demonstrated. 3,3′-Diindolylmethane modulates autophagy activation by miR-30e-ATG-5 in BGC-823 and SGC-7901 cells (Ye et al., 2016). Pectolinarigenin, a natural flavonoid present in *Cirsium chanroenicum*, downregulated PI3K/AKT/mTOR pathway *via* G2/M phase cell cycle arrest, apoptotic, and autophagic cell death in human GC cells (Lee et al., 2018). Flux analysis of autophagy and increase in the level of LC-3-II revealed induction of autophagy by the tuber of *Amorphophallus konjac*. G0/G1 phase cell cycle arrest has been detected by flow cytometry. Chen et al. determined apoptosis- and autophagy-inducing effects of kangfuxin, an organic extract of *Periplaneta americana* Linnaeus. (Blattidae), against SGC-7901 cell line (Chen et al., 2017). Proteins that mediate ER stress–mediated apoptosis including glucose-regulated protein 78 (GRP78), C/EBP-homologous protein (CHOP), and caspase-12 have been greatly upregulated in the group treated with kangfuxin. In addition, the LC-3-I/LC-3-II ratio and expression levels of beclin-1 were also higher in the kangfuxin group. Also, 3,3′-diindolylmethane, derived from cruciferous vegetables, increased the ATG-5 expression and LC-3 in GC cells in addition to decrease miRNAs-30e level (Ye et al., 2016). Besides, perillaldehyde, isolated from *Perilla frutescens*, increased AMPK phosphorylation, leading to autophagy *via* beclin-1, LC-3-II, cathepsin, p53, and caspase-3 in tumor xenograft model of GC in MFC mouse as well as GC9811-P human GC cells (Zhang et al., 2018b). Danggui-Sayuk-Ga-Osuyu-Saenggang-Tang (DSGOST), a traditional Chinese medicine, stimulates autophagy by triggering AMPK/ULK1 signaling, consequently activating EMT and exosomes to increase chemoresistance, and therefore, one of the mechanisms of GC resistance to DSGOST is survival, promoting autophagy. Using compound C, a well-known suppressor of AMPK, to impede DSGOST-mediated autophagy and diminish drug resistance, promotes killing of GC cells and decreases their drug tolerance (Kim et al., 2018a). In addition, natural plant components of *Saussurea lappa* Clarke, *Dioscorea nipponica* Makino, and *Melandrium firmum* have been found to induce antiproliferative and apoptotic functions (Rahman et al., 2013; Rahman et al., 2014; Rahman et al., 2015). Latcripin 1 (LP1) was found to arrest S-phase cell cycle as well as decrease matrix metalloproteinase 2 (MMP-2) and MMP-9 expression with induction formation of autophagosomes by AKT/mTOR pathway against GC cell lines SGC-7901 and BGC-823 (Batool et al., 2018). Allicin has been found to increase autophagy in human GC cell MGC-803, BGC-823, and SGC-7901 cells *via* regulation of p38 signaling (Zhang et al., 2015). Kaempferol, a flavonoid isolated from fruits and vegetables, induces autophagic cell death through IRE1-JNK-CHOP activation in response to ER stress in human GC cell lines (Kim et al., 2018b). Numerous phytochemicals and their response in GC by autophagy signaling are presented in Figure 5. Therefore, autophagy induction by phytochemical might possibly be targeted as a potential therapeutic approach to control GC.

**TABLE 2:** Phytochemicals and their effects on gastric cancer *via* autophagy pathway.

Compounds	Experimental model/cells	Dose/duration	Autophagy mechanism	References
N-butylidenephthalide	AGS	25, 50, 75 μg/mL; 24 h	↑REDD1	Liao et al. (2018)
			↓mTOR	
Perillaldehyde	Xenograft model of gastric cancer	100 mg/kg per day	Beclin-1, LC-3-II, cathepsin, p53-mediated autophagy	Zhang et al. (2018b)
Terpenoid	AGS, MKN-45, KATO III	10, 20, 30 μM; 24 h	↑p-JNK, p-p38, p-AMPK, Bax, cyt c, caspase-3, c-PARP1, LC-3-II	Chun et al. (2014)
			↓p-ERK, p-AKT, p-mTOR, NF-κB, COX-2, cyclin D1, VEGF, Bcl-2, Bid	
Kangfuxin	SGC-7901	0.1, 1 μg/mL; 48 h	↑GRP78, CHOP, caspase-12, LC-3-II/LC-3-I, Bax	Chen et al. (2017)
			↓Bcl-2	
Rottlerin	SGC-7901, MGC-803	2 ,4, 8 μM; 24 h	↑LC-3-II	Song et al. (2018)
			↓mTOR, Skp2	
Allicin	Human gastric cancer cell MGC-803, BGC-823, and SGC-7901	1 μg/mL	Increase expression of p38 and autophagy	Zhang et al. (2015)
Evodiamine	SGC-7901	10 µM	Activates beclin-2 and autophagy	Rasul et al. (2012)
Pectolinarigenin	AGS and MKN-28	50 and 100 μM	PI3K/AKT/mTOR signaling	Lee et al. (2018)
3,3′-Diindolylmethane	BGC-823 and SGC-7901	60 μM	miR-30e-ATG-5 modulating autophagy	Ye et al. (2016)
Latcripin 1	SGC-7901 and BGC-823	30, 60, and 90 μM	ATG-7, ATG-5, ATG-12, ATG-14, and beclin-1 induction autophagy	Batool et al. (2018)
Kaempferol	Human GC cell lines (AGS, SNU-216, NCI-N87, SNU-638, and MKN-74)	50 μM	Activation of the IRE1-JNK-CHOP–mediated autophagy	Kim et al. (2018b)

## 8 Role of Autophagy Activators and Inhibitors on GC

There are no clear evidences whether autophagy has a cancer-promoter or a cancer-suppressor function in GC. It has been found that autophagy plays an important role in the human cancer patient who suffers chemoresistance, and development of molecule that acts as activator or inhibitor for autophagy may be an open novel resource to treat GC (Mele et al., 2020; Mendes et al., 2021). CQ and hydroxychloroquine (HCQ) are approved by the Food and Drug Administration for clinical application, which can be blocking degradation and the fusion step of autophagosome as a result of regulating autophagy to illustrate the dual role of autophagy in cancer. At present, multiple types of tumors are treated by CQ and HCQ separately or mixed with chemotherapy (Lin et al., 2017). Moreover, it has been found that abnormal expression of autophagy genes may cause some cancer-related pathology. Kim et al. (2020) discovered that genipin, derived from *Gardenia jasminoides*, can boost p53, a tumor-suppressor protein p53 and DRAM, trigger apoptotic cell death and autophagy, and improve the susceptibility of AGS and MKN45 GC cell lines to OXA by increasing p53 and DRAM (Kim et al., 2020). According to Xu et al. (2018), tanshinone IIA can diminishes the expression of MRP1 and impede adriamycin efflux (ADM) (Mirzaei et al., 2021). The mixture of ADM and tanshinone IIA may also increase the sensitivity of GC cells to ADM by inducing autophagy and boosting the death of the cells (Xu et al., 2020a). TCM licorice contains a compound known as liqueritin that promotes beclin-1 expression and reduces p62 expression, which activates autophagy (Zhao et al., 2021). DSGOST, a traditional Korean herbal remedy induced by several chemotherapy medications, is often utilized to activate the AMPK/ULK1 pathway, increasing autophagy flux, inducing autophagy and apoptosis, and increasing the sensitivity of the GC cell lines AGS and SNU-638 (Kim et al., 2018a). Furthermore, excessive or aberrant activity of autophagic may activate cytotoxicity in addition to contributing to intracellular components’ improper degradation, which is essential for maintaining cancer cell survival in GC cells (Qian and Yang 2016). Researchers have discovered that the herbal remedies cucurbitacin B and phloretin have anticancer properties and reverse chemotherapy resistance in recent years (Choi 2019; Fabiani 2020). Several investigations on their mechanisms of action have revealed that cucurbitacin B inhibits CIP2A (cancerous inhibitor of protein phosphatase 2A), which then reactivates PP2A (protein phosphatase 2A), hence increasing PP2A-dependent mTORC1 inactivation and decreasing PP2A-independent mTORC1 activation (Liu et al., 2017). Phloretin suppresses ERK1/2 and MAPK p38 phosphorylation and enhances LC-3B II and beclin-1 expression, thereby initiating autophagy and increasing the susceptibility of GC cells to ADM *in vitro* (You et al., 2020). The presence of irregular glycosylation has long been considered a warning sign of cancer, and this has been linked to tumor growth, progression, metastasis, and resistance to chemotherapy (Wang et al., 2020). In GC cells, 5-FU was found to induce cell proliferation arrest, as well as autophagic cell death *via* beclin-1 upregulation, which significantly enhanced autophagy and reduced cancer cell growth. Therefore, inducing autophagy may efficiently control GC (Yang and Pan 2016).

Numerous therapeutic can promote protective autophagy in cancer cells, thereby preventing cancer cells from undergoing drug-induced apoptosis. One study demonstrated that oxaliplatin can partially antagonize apoptotic cell death in GC MGC803 cells that protect autophagy-induced cell death (Xu et al., 2011). Tunicamycin was first discovered as a natural antibacterial and anticancer chemical because it is an effective glycosylation inhibitor (Wu et al., 2018a). Inhibiting N-glycosylation with tunicamycin boosts ER stress and autophagy while increasing the susceptibility of GC cells to ADM and VCR (Wu et al., 2018a). In contrast, OGT inhibitor–mediated autophagy was significantly attenuated by 3-MA, a blocker of autophagosome formation, however, when pretreated with CQ (Rahman et al., 2019; Rahman et al., 2020a). Indomethacin, a well-known NSAID, has been used successfully as a coadjutant in the development of anticancer medicines (López-Contreras et al., 2020). In AGS cells, indomethacin increases OXA-induced cell death, increases p62 and NBR1 accumulation, impairs lysosomal activity, and inhibits autophagic destruction (Vallecillo-Hernández et al., 2018). DDP reduces MALAT1 expression, whereas propofol increases the inhibitory effects of miR-30e on ATG5 and autophagy, making GC highly susceptible to DDP both *in vitro* and *in vivo* (Ashrafizadeh et al., 2020; Tabnak et al., 2021).

## 9 Conclusion and Prospects

In tumor progression, autophagy plays a complicated task and diverse consequences, depending on what type of tumor and its phases. The role of autophagy has theoretical as well as clinical significance to maintain cellular homeostasis. In the initial stages of malignant transformation or cancer formation, autophagy appears to restrict tumor growth, but in the late stages, autophagy seems to enhance tumor survival as well as tolerance to chemotherapy. Several factors may control the intracellular autophagy level, which determines the effectiveness of antitumor therapies based on autophagy modulation in GC. While the PI3K and mTOR regulatory systems have been proven as major signaling routes governing autophagy, alternative autophagy-related mechanisms (p53, MAPK, or PTEN) must be investigated in further studies. The importance of miRNA and *Helicobacter pylori* in the control of autophagy in GC regulation is rapidly being recognized, while autophagy stimulators and blockers have obtained considerable clinical testing results in the treatment of GC, which has potential therapeutic approaches as well. Nevertheless, the dual role of autophagy therapy in GC should also be addressed. Many investigations, from the other side, have shown that autophagy and apoptosis can coexist or happen in consecutive order, and they can combine to modulate cancer regulation and management. Because of the extreme variation and unclear source of antitumor drug resistance, which causes inefficient medication and poor diagnosis in patients with progressive of GC, current research has summarized the complicated interaction involving autophagy and chemotherapy protest in GC. Although rapid recognition and monitoring of GC are critical stages in the therapeutic process, as a result, associated proteins and ATGs are accused of being engaged in autophagy methods and are predicted to get more new targets and diagnostic markers for GC treatments. Even more research into autophagy indicators may offer a new option for prognostic indicators and medicinal objectives for GC. Furthermore, more data suggest that too many organic ingredients can cause chemotherapy tolerance by controlling signal transduction. As a result, natural substances, either alone or in conjunction with autophagy modulators and/or chemotherapeutic medicines, might have a beneficial impact on drug-resistant malignancy in GC. However, additional research is required to discover molecular mechanisms and particular objectives, as well as to validate the efficacy and safeness of these treatments in medically significant in GC cancer models. It should be confirmed how autophagy functioning is controlled variably in GC, or which variables induce tissue-specific suppression and/or stimulation of autophagy.
